# Supplementary material for: Indistinguishable mitochondrial phenotypes after exposure of healthy myoblasts to myalgic encephalomyelitis/chronic fatigue syndrome or control serum
Source: PLoS One. 2026 Feb 3;21(2):e0341334. doi: 10.1371/journal.pone.0341334 (PMC12867253; doi:10.1371/journal.pone.0341334)
Supplement: S1 File — (DOCX) [file pone.0341334.s001.docx]

Supplementary figures:


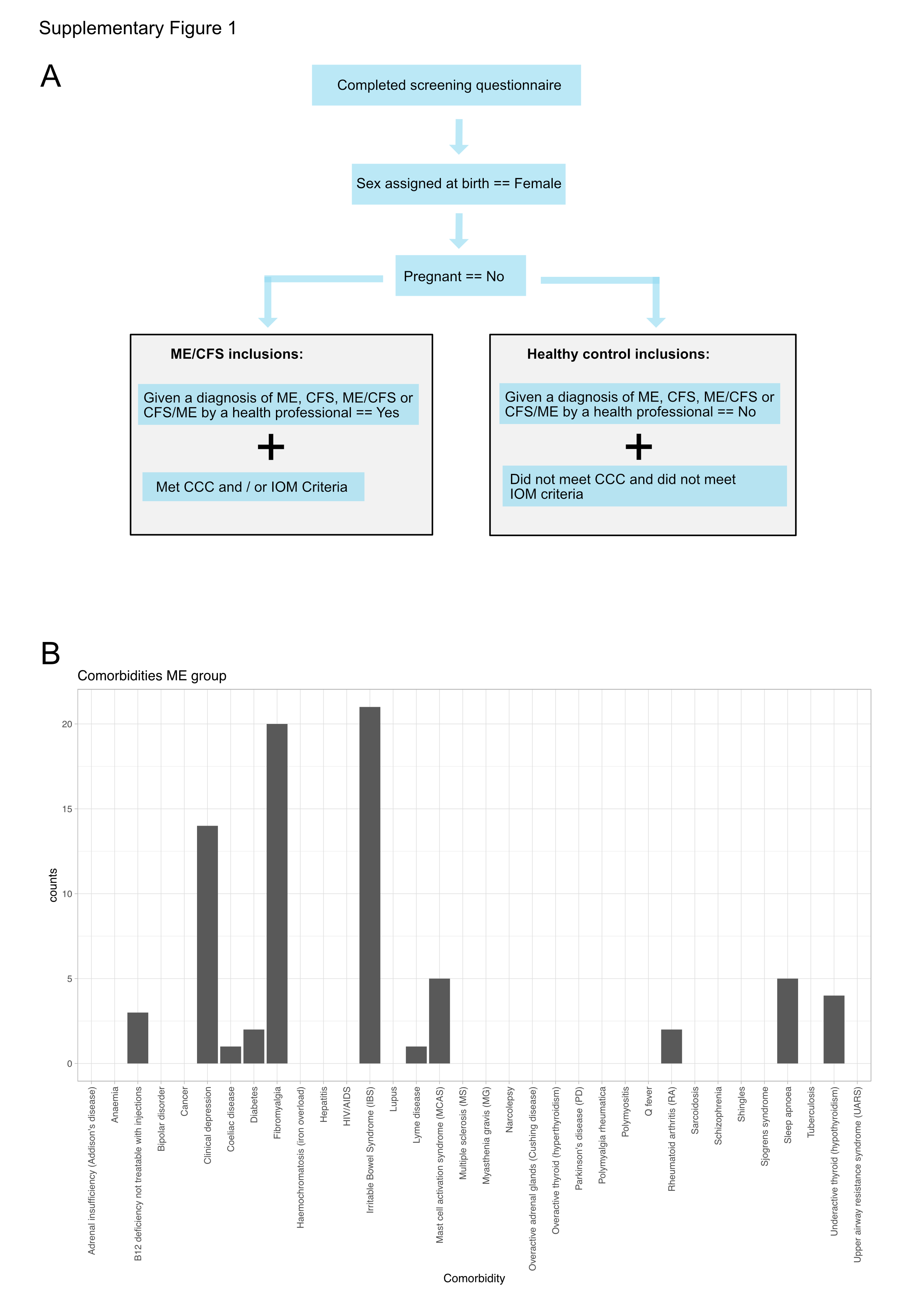


Supplementary Figure 1: A) Flow chart of screening process for pwME and controls. B) Comorbidities reported by pwME in our study.


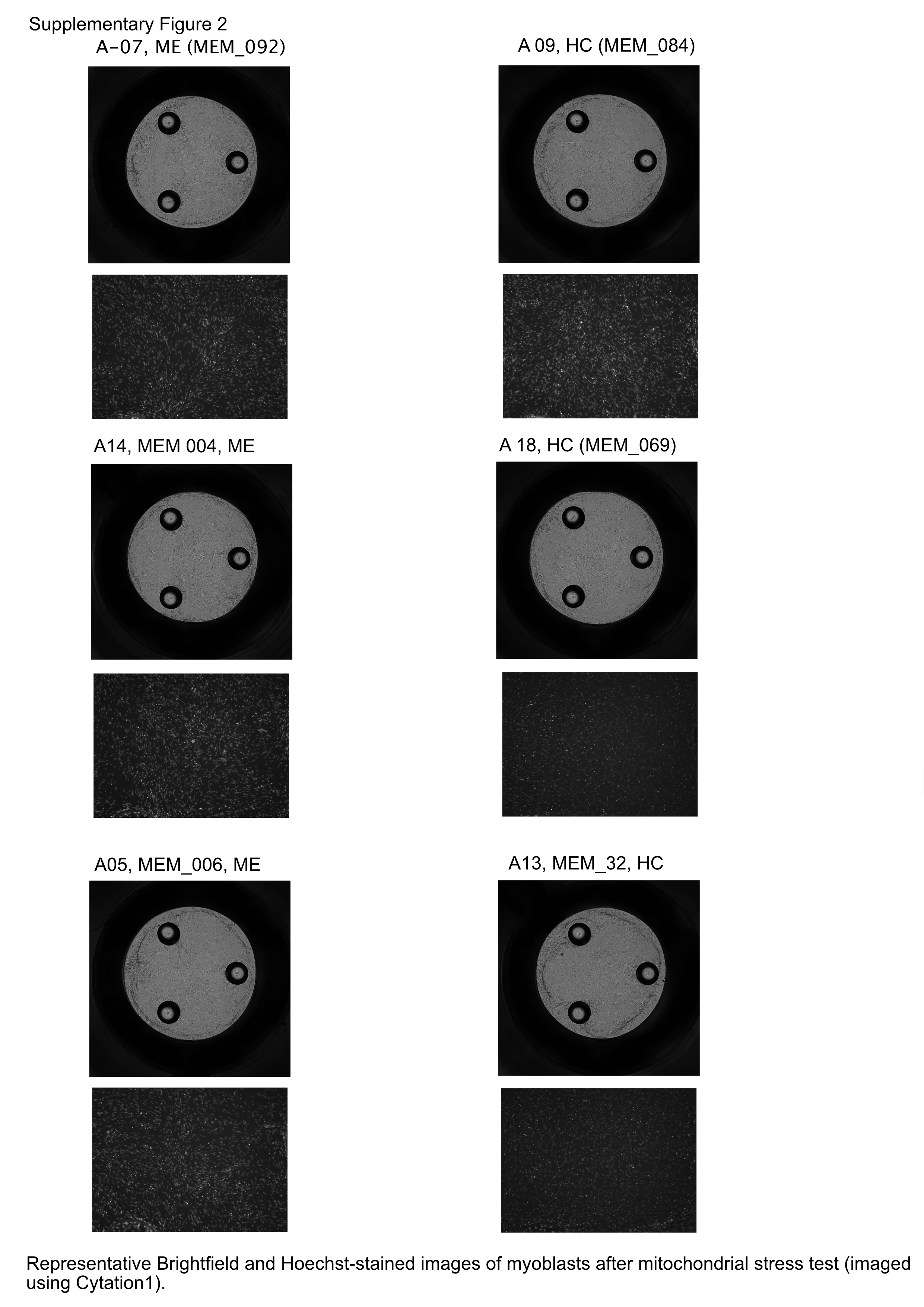


Supplementary Figure 2: Representative Brightfield and Hoechst-stained images of myoblasts after mitochondrial stress test (imaged using Cytation1)


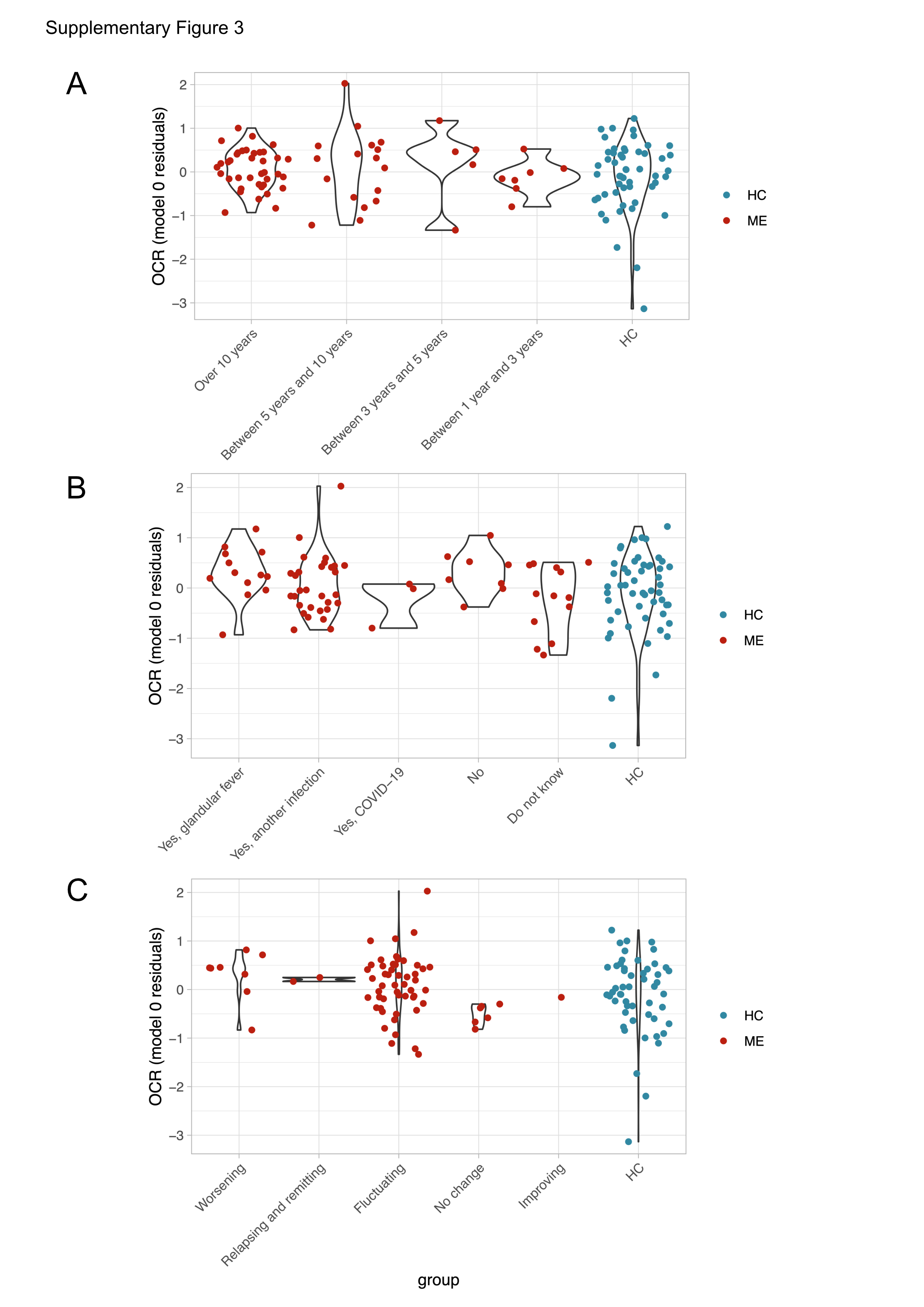
Supplementary Figure 3: A) OCR residuals at maximal respiratory capacity averaged by individual, and stratified by disease duration B) trigger type, and C) illness course.


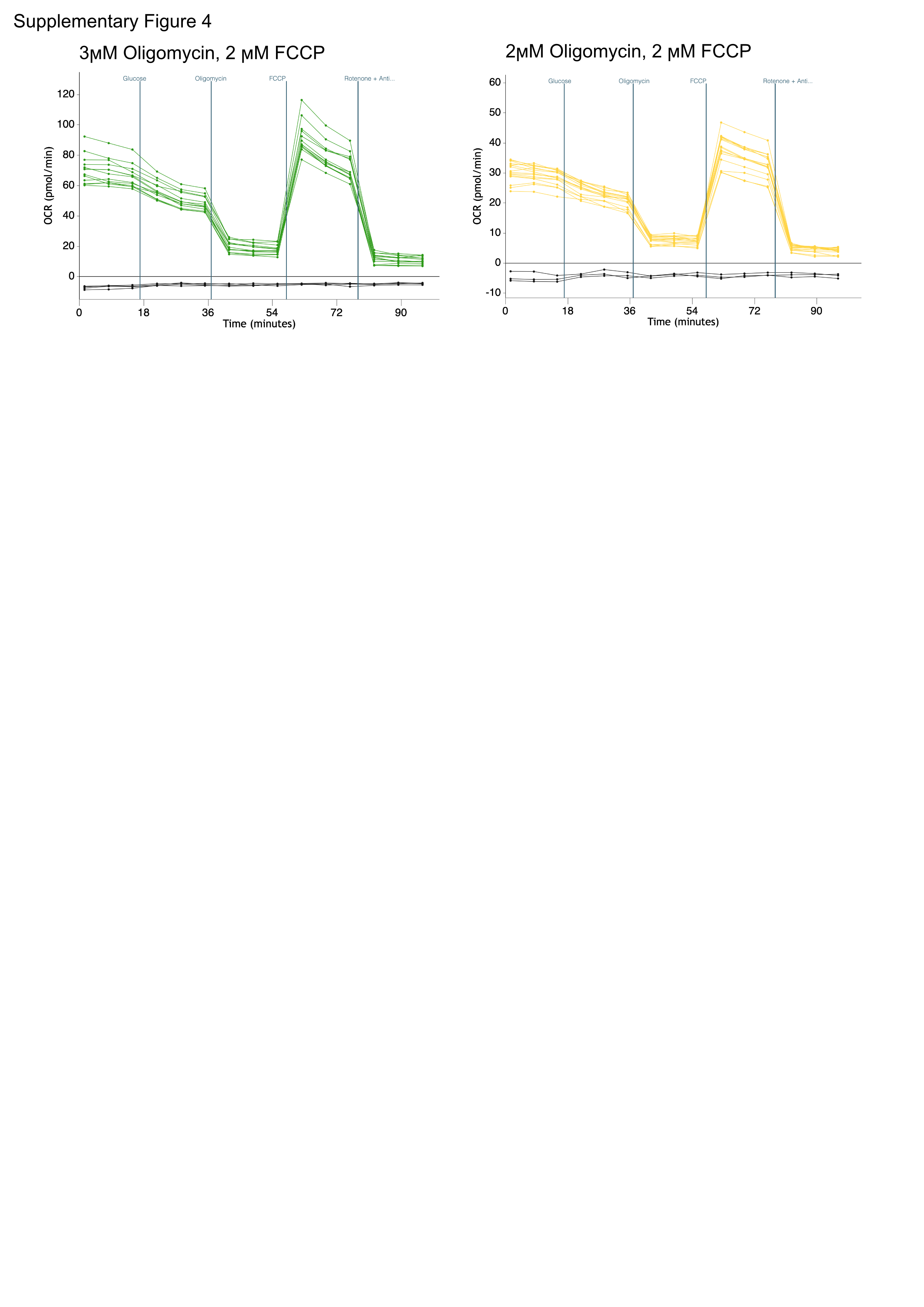
Supplementary Figure 4: Testing Oligomycin concentrations, on myoblasts treated with FBS.
