# Supplementary material for: Indistinguishable mitochondrial phenotypes after exposure of healthy myoblasts to myalgic encephalomyelitis/chronic fatigue syndrome or control serum
Source: PLoS One. 2026 Feb 3;21(2):e0341334. doi: 10.1371/journal.pone.0341334 (PMC12867253; doi:10.1371/journal.pone.0341334)
Supplement: Attachment — Submitted filename: response_to_reviewer_25_09_25.docx [file pone.0341334.s007.docx]

Dear Editor,

Thank you for taking the time to consider our manuscript and for your helpful comments and feedback.

In light of your review, we have revised key sections in the Introduction and Discussion sections to better contextualise our results within the body of ME literature and to highlight limitations of examining cellular rather than molecular phenotypes. We have expanded on what future studies might explore, and further clarified the contribution that our well-powered study has made to an evidence base that often lacks rigour and reproducibility.

We here address each of your 3 points in turn:

1) Enhancing the mechanistic depth of the study by assessing gene expression for glycolysis/OXPHOS components.

We agree with the value of performing additional experimental work to explore possible mechanisms of ME serum-induced mitochondrial phenotypes beyond the cellular phenotypes reported in our study. The limited funding and sample material available to us, however, makes this additional work impossible. The cost of performing RNA sequencing or qPCR of key glycolysis and OXPHOS genes on 120 cell cultures exposed to ME or control serum far exceeds the remaining budget on this charity-funded grant.

We agree that had we observed any differences in cellular phenotype between our disease and control groups then this would have been a particularly informative additional work package. It is possible that metabolic reprogramming is occurring and that changes at a *molecular level* might be observed in the myoblasts with methods such as gene expression quantification. However, this question lay beyond the scope of our pre-registered study which was focussed, instead, on changes in phenotype at the *cellular level*. You have raised an important point and hence we have now commented on this limitation of our work in the Discussion section.

2) Compensatory or maladaptive metabolic responses measured by orthogonal assays such as studying cell free RNA.

Examining the possibility of compensatory/maladaptive metabolic responses in ME is certainly of interest to the field. Our assay, however, was intended instead as a study system that could screen for differences in serum factors between ME and controls, rather than examining mechanisms of metabolic dysfunction in ME directly. The study by Gardella et al 2025 (<https://doi.org/10.1073/pnas.2507345122>) that you pointed to (which, notably, was published after we submitted our paper to your journal - 11/08/2025), provides an interesting hypothesis-generating approach to identify novel biomarkers for ME using cell free RNA. Their findings identify that certain cell free RNA transcripts – including a significantly lower percentage of mtRNA in those with ME - might be used to distinguish healthy control and pwME plasma using machine learning. While their results suggest that metabolic dysfunction may be observed in the blood from people with ME, we do not expect cell free RNA in the blood, which originates mainly from dying platelets, red blood cells and leukocytes, to be directly relevant to the metabolic adaptations of the cultured myoblasts exposed to ME serum in our study. Unfortunately, performing cell free RNA quantification is again not feasible due to our limited remaining research budget and available serum, and because studies exploring cell free RNA have demonstrated low reproducibility and high technical and biological variation (<https://doi.org/10.1186/s40364-022-00409-w>).

3) Strengthening the conclusions and improving the translational relevance of the findings.

This work demonstrates that using *in vitro* models of examining cellular phenotypes of healthy myoblasts exposed to serum from people with ME is unlikely to lead to translational findings. This knowledge is critical for the ME field in which translational research remains in its infancy. Establishing a firm evidence base is crucial and without the publication of null results and replication studies, future research will continue to follow fruitless lines of enquiry.

Finally, we wanted to emphasise why we believe our paper merits publication in PLOS One and fits the scope of your journal. The PLOS One website states: “We evaluate research on the basis of scientific validity, strong methodology, and high ethical standards—**not perceived significance**. Multidisciplinary and interdisciplinary research, **replication studies, negative and null results are all in scope**. We also publish Registered Reports and Protocols” (<https://journals.plos.org/plosone/s/journal-information>). Unlike many other journals that are concerned only with the perceived novelty of results, PLOS One’s emphasis on rigour and good scientific practice regardless of negative results is a key reason why we chose to submit our work to your journal. It is our conviction that the scientific rigour with which our research was carried out, including **preregistration** of our study on the Open Science Foundation (<https://osf.io/qwp4v>), makes it well-suited to the scope of PLOS One, despite the negative findings.

Thank you for considering our manuscript and we look forward to hearing from you soon.

With very best wishes,

Audrey Ryback
