# Supplementary material for: Indistinguishable mitochondrial phenotypes after exposure of healthy myoblasts to myalgic encephalomyelitis/chronic fatigue syndrome or control serum
Source: PLoS One. 2026 Feb 3;21(2):e0341334. doi: 10.1371/journal.pone.0341334 (PMC12867253; doi:10.1371/journal.pone.0341334)
Supplement: Attachment — Submitted filename: Response_to_reviewers_20_12_25.docx [file pone.0341334.s008.docx]

We would like to thank all three reviewers for their constructive feedback and suggestions, and have addressed their points in turn. Please note that line numbers referred to in this document correspond to the line numbers in the version of the manuscript with tracked changes.

Reviewer #1: This study aimed to replicate findings from Fluge et al. (2016), which suggested that serum from individuals with Myalgic Encephalomyelitis/Chronic Fatigue Syndrome (ME/CFS) could increase mitochondrial respiratory capacity in healthy myoblasts. Using a larger sample size (67 ME/CFS patients and 53 healthy controls), researchers treated cultured myoblasts with serum and conducted over 1,700 mitochondrial stress tests using a Seahorse Bioanalyser. Contrary to the original findings, they found no significant differences in oxygen consumption rates at maximal respiratory capacity between the ME/CFS and control groups. These results challenge the hypothesis that ME/CFS serum contains factors that alter mitochondrial function in vitro, suggesting limited utility for this approach in developing diagnostic tests.
Strengths: The study is commendable for its careful experimental design, robust methodology, and transparent discussion of potential biases and limitations. Additionally, the authors provide a clear rationale for their approach and acknowledge the constraints of their experimental system.

Suggestions for Improvement: It would have been beneficial to include microscopic monitoring of the assays, with representative cell images and viability dye staining/counts before and after treatment. This would help assess potential morphological changes or cytotoxic effects that might not be captured by metabolic measurements alone.

We agree that microscopic monitoring of cells in response to serum treatment would be an interesting avenue of investigation. Showing morphological changes to the cells that would be statistically representative over the complete course of the experiment lay beyond the scope of this study’s aims. In the revised submission we include Brightfield and Hoechst-stained images of cells after treatment to illustrate that cells remained intact: see additional Figure: Supplementary Figure 2, discussed in the manuscript (see lines: 294-296-highlighted in yellow). We agree that it was important that we accounted for potential cytotoxic effects of serum exposure. This was why we included cell counts as a covariate in our analyses (see “model 1”).

Weaknesses and Considerations: A key limitation lies in the heterogeneity of the ME/CFS patient population, which may obscure subtle serum-induced effects. The wide variability in OCR and ECAR values across individual samples further complicates interpretation. Although the study increased the sample size compared to Fluge et al. (2016), the low representation of severely affected patients may limit the generalizability of the findings, which appear restricted to mild/moderate cases. While stratification by severity did not yield significant differences, it would be valuable to know whether the authors conducted post hoc stratified analyses based on other clinical or biological traits or employed dimensionality reduction techniques such as PCA to explore latent patterns.

We agree that ME symptoms and severity are heterogeneous, a fact that always presents a challenge when studying ME. Specifically, the low representation of severely affected patients may have limited our statistical power to replicate the findings from Fluge et al. (2016). Upon recommendation by the reviewers, we have conducted post hoc stratified analyses of OCR with different disease features (disease duration, trigger, and illness course). We found no evidence for subgroup-specific serum effects based on these variables (see lines 448-453, highlighted in yellow, and new Supplementary Figure 3). We have chosen not to employ dimensionality reduction techniques in this instance, because the sample size for individual clusters would be too small to be informative.

Data Presentation: In Figures 4D and 4G, the greater dispersion of values in batch 2 for both study groups is noticeable. A brief commentary on potential causes (such as batch effects, sample handling, or donor variability) would help readers better understand this discrepancy and its implications for data interpretation.

We have added a brief comment, as suggested (see lines: 441-443, highlighted in yellow)

In Figure 4, it appears that 1–2 data points in the healthy control (HC) group may be outliers. It would be helpful if the authors addressed this, either by discussing their impact or clarifying whether any statistical treatment was applied.

All exclusions were decided prior to unblinding. Outliers were excluded if measurements were deemed unreliable due to improbable cell counts (cell counts below 8000 and above 35000), or measurements taken at maximal respiratory capacity were above Q3 + 1.5 x IQR or below Q1 – 1.5 x IQR (see Methods, lines 213-216). However, the scatterplots and violin plots in Figure 4 clearly indicate that the spread of the data is very similar between both groups.

Tables 1 through 3 could be consolidated into a single comprehensive table to improve readability and reduce redundancy.

Agreed. We have collapsed tables 1-3 into a single table (see “Table 1”), as suggested.

Figures should include exact p-values, in addition to the current "n.s." (not significant) labels. Consistency in formatting across all figure labels would enhance clarity.
There is a typographical error on line 203.

Thank you. We have added the p-values to all figures as suggested, and corrected the typographical error.

Reviewer #2: The manuscript presents a well-designed, preregistered replication of the study by Fluge et al. (2016), evaluating the effects of ME/CFS patient serum on mitochondrial function in cultured myoblasts. The study is technically solid, uses a sufficiently powered sample, and applies rigorous statistical methods with proper control of plate effects and randomization. The authors should be commended for their transparency, detailed reporting, and for publishing negative results—an important and often underrepresented aspect of the scientific process. These features make the paper a valuable contribution to the ME/CFS field, which greatly benefits from efforts to test reproducibility under controlled conditions.

However, while the experimental rigor is strong, the narrative emphasis of the paper leans heavily toward discrediting the results of Fluge et al., rather than leveraging this replication outcome to address broader questions about biological variability and cohort standardization in ME/CFS research. A more balanced framing would increase the paper’s constructive impact and relevance beyond a single prior study.

To be specific, the study convincingly shows that no serum-driven mitochondrial phenotype was observed under the specific experimental and cohort conditions tested here. Yet, direct comparability with Fluge et al. remains limited due to several biological and clinical differences between cohorts:

(1) Sex distribution: This study includes only women, while Fluge et al. analyzed both sexes and observed stronger effects in females.
(2) Disease severity and duration: The original cohort contained mostly moderate-to-severe, long-standing ME/CFS cases, while the present cohort is milder, and disease duration is not clearly reported.

(3) Age, medication, fasting state, and comorbidities: These variables are not fully described and may affect circulating metabolites and mitochondrial behavior.
These distinctions suggest that both studies may not be addressing precisely the same biological question, even if the technical protocol is similar. Therefore, it would be more accurate to interpret the current results as indicating no detectable effect within this specific cohort, rather than as a categorical refutation of previous findings.

We agree that biological or clinical differences may exist between the cohorts in the two studies, particularly pertaining to disease severity. Nevertheless, we expect a biological signal with a large effect size (Cohen’s D=1.32), as found in the original study, to be observable upon replication in a study with our statistical power, if it were a generalisable feature of ME. Furthermore, to address the specific points raised:

(1) We intentionally selected only female participants to increase our power to detect any differences. While Fluge et al. (2016) stratified their cohort for performing metabolomic analysis, they did not perform a sex-stratified analysis for the sub-cohort used in the Seahorse experiments and did not report stronger effects of ME serum on OCR in females.
(2) All of our participants were screened using the DecodeME questionnaire and had a clinical diagnosis of ME, and met CCC and/or IoM diagnostic criteria. All of our cases had a disease duration of at least 1-3 years and most of our cases had a disease duration of more than 10 years (see new Supplementary Figure 3). Our study provides greater detail on the cohort characteristics beyond that provided for the subcohort used to perform the Seahorse experiments in Fluge et al.
(3) We explored the relationship between OCR and age in Figure 5C and found no statistically significant correlation (R squared value = 0.0). Information about comorbidities can be found in Supplementary Figure 1B. While we did not include information about fasting status or medications, neither did Fluge et al. (2016) for the sub-cohort on which they performed the experiments that we sought to replicate.

Furthermore, the cohort in Fluge et al. (2016) is subject to potential batch effects: ME cases were collected and stored in 3 batches, whereas healthy controls were collected in 2 further batches, some of which were several years apart:

*“62 healthy controls were recruited from blood donors at Haukeland University Hospital, with blood samples taken in* ***2012****. 40 healthy controls were recruited from the staff at the Department of Oncology, Haukeland University Hospital, with blood samples taken in* ***2015****. […]”*

Meanwhile, the ME cases were recruited from three separate clinical trials:

*“The majority of samples from ME/CFS patients were harvested in late 2014 and 2015 (181 samples from the “RituxME” and “CycloME” trials). The remaining 19 samples were collected in 2010 (in the KTS-2-2010 trial).”*

As far as we can see, Fluge et al. (2016) did not account for these batch effects in the Seahorse experiments described in the paper and did not specify which batches the cases and controls originated from. Furthermore, all 102 healthy controls were non-fasting, whereas 47 of the ME cases fasted overnight prior to biobank sampling, while 153 did not. They did not specify whether the 12 ME samples used in their experiments were taken from fasting or non-fasting individuals.

In our study both controls and cases were sampled in each batch and the two sampling batches were acquired only 3 months apart, so that the effect of different storage times and other batch effects were minimised.

I encourage the authors to revise the Discussion to highlight this key point and to extract a broader methodological message for the field: the necessity of standardized and well-documented cohort characterization (sex, age, disease duration, severity, metabolic state, and preanalytical conditions). Such harmonization would greatly enhance reproducibility and interpretability across future ME/CFS studies.

We agree that there is a need for standardised and well-documented cohort characterisation across the field, and have added this point to the Discussion section (lines 539-541, highlighted in yellow).

In summary, this manuscript has clear value due to its methodological strength, transparency, and negative results. Reframing the discussion from a refutation to a constructive call for methodological standardization would strengthen the scientific and conceptual contribution of this work.

Reviewer #3: Introduction

You have introduced the term “people with56 ME (pwME).” Please not that The acronym “pwME” is not universally recognized and may confuse readers unfamiliar with the convention. It is not universal to describe people suffering from other conditions (heart failure etc.) as people with XX. Using such approach might further distant the general public view of the field of ME/CFS from rest of biologically-based severe conditions.

While we share your concern about stigmatisation of ME, and we certainly do not wish to distance ME from biologically-based severe conditions, we chose to use the term “people with ME” (pwME) only after it was recommended by individuals with lived experience of ME. We further involved a patient and public involvement panel in this project who reviewed the manuscript and who did not flag this terminology as a concern.

Methods
“Sera from pwME and HC were collected between 27/11/2023 - 23/02/2024 across two
rounds of sampling over two weeks in November–December 2023 (“batch 1”), and
three weeks in February 2024 (“batch 2”) (Table 1).”
Do You think that it might affect results obtained? Please describe it as a potential limitation.

We included sensitivity analyses exploring batch effects in Figure 5D and Figure 5G. We note that in Fluge et al. (2016) batch effects were likely to be more substantial than ours. This is because their patient and control samples came from different sources, some which were collected over a number of years. They do not specify which batches their samples were obtained from, whereas the ME cases and controls we recruited were sampled in both of our batches. We further performed a sensitivity analysis that included sampling batch as a covariate and demonstrated no statistically significant effect of batch on OCR (lines 432-448).

“Due to the female preponderance of ME (3) and to reduce heterogeneity, all study participants were female. “ Please describe it as a potential limiting factor of the study

Cohort heterogeneity is a limitation of many studies of ME, including ours. We see the restriction to females as a strength of our study, rather than a limitation, because it minimises one source of potential heterogeneity. Including both sexes would have reduced our statistical power to detect differences, particularly since there is no existing evidence of male-specific effects of ME serum on mitochondrial function. By focussing only on female ME cases, we increased our statistical power.

“People with ME met the Canadian Consensus Criteria (CCC) and/or the Institute of Medicine (IoM) diagnostic criteria and reported a diagnosis of ME by a healthcare professional. Healthy controls did not meet the CCC or IoM criteria according to their screening survey responses and did not report any of the 21 active comorbidities screened for by the DecodeME screening questionnaire (13).” How many met IOM and how many met CCC? How ME patients and HCs were recruited? Please describe, using flowchart might help

We have now added this information to the methods (lines 120-121, text highlighted in yellow) and a flowchart to Supplementary Figure 1A describing the screening criteria.

Discussion:
“Consequently, our study’s results do not support the hypothesis that ME sera impact on healthy myoblast mitochondrial phenotypes differently from healthy control sera.” Can You cite previous studies on blood cell‐based diagnostic test in CFS, that would effectively delineate patients vs controls? What variables were taken into account in those studies?

We cite in the Introduction several studies that found differences between cells exposed to ME or control serum or plasma (lines 69-80). We focussed on citing and explaining these studies as they were the closest methodologically (predominantly serum swap) and closest in terms of area of biological investigation (predominantly mitochondrial biology). Three additional studies have claimed evidence for blood cell based diagnostic tests in ME/CFS ([Esfandyarpour et al. 2019](doi:%2010.1073/pnas.1901274116), [Xu et al. 2023](https://pmc.ncbi.nlm.nih.gov/articles/PMC10602530/), [Hunter et al. 2025](https://link.springer.com/article/10.1186/s12967-025-07203-w)). The fundamental limitation that these papers share is that they are based on modest sample sizes and have not been replicated. As our paper demonstrates, we cannot take the results from single small scale studies in ME as definitive. Therefore, we do not think there are at the present time any studies that we can cite that definitively diagnose patients from controls, even if some methods may show promise.

“A further limitation of our study is that participants with ME may not have been
experiencing post exertional malaise (PEM) on the day of sampling. “ Can You describe briefly previous studies on how physical activity affects mitochondria in CFS patients?

[Germain et al. (2025)](https://www.sciencedirect.com/science/article/pii/S1535947625005663) subjected pwME and matched sedentary controls to two maximal cardiopulmonary exercise tests separated by a 24-h recovery period. They observed an upregulation of glycolysis/gluconeogenesis in proteomic data from plasma, suggestive of mitochondrial stress and impaired immune recovery from exercise. However, only patients with less severe ME would be able to participate in such a study. In [Appelman et al. (2024)](https://www.nature.com/articles/s41467-023-44432-3) the authors report that skeletal muscle structure from people with Long Covid is associated with a lower exercise capacity. They observe that after performing a cardiopulmonary exercise test, local and systemic metabolic disturbances worsen and they find severe exercise-induced myopathy and tissue infiltration of amyloid-containing deposits in skeletal muscles. Whether these findings are generalisable to ME is not known. How physical activity affects mitochondria in ME in general remains poorly understood in the current literature and lies beyond the scope of the paper.

The cohort includes mostly mild-to-moderate ME/CFS cases, whereas Fluge et al. studied severe/very severe patients. While the authors acknowledge this, they could more explicitly discuss whether their null result might be due to disease heterogeneity rather than a true refutation of the original finding. Please add a paragraph in the Discussion exploring whether ME/CFS subtypes (e.g., based on PEM severity, onset type, or immune profile) might differentially affect serum bioactivity. Cite recent work (e.g., from DecodeME or NIH intramural studies) suggesting ME/CFS is not a monolithic condition.
The authors note that participants were not necessarily experiencing post-exertional malaise (PEM) at blood draw, which could mask transient serum factors. What kind of a future study design where blood is collected before and after a stressor could be done? What limitations such study would have, including ethical concerns?

We agree that disease heterogeneity limits the ability to compare findings between our cohort and the cohort in Fluge et al. (2016). We have now conducted post hoc stratified analyses of OCR with different disease features (disease duration, trigger, and illness course) and find no evidence for subgroup-specific serum effects based on these variables (see: Supplementary Figure 3, Results section (lines 448-453, highlighted in yellow). See also our response to Reviewer #1, above. Additionally, in the Discussion we have now also addressed the potential for future study designs that would capture or account for the effects of PEM (549-553, highlighted in yellow).

Please discuss whether alternative assay platforms might be better suited for detecting small effect sizes in future work.

In the introduction section (lines 77-80), we discuss findings from [Beentjes et al (2025)](https://pubmed.ncbi.nlm.nih.gov/40537675/) that employed alternative approaches for biomarker identification on a large dataset enabling the detection of small effect sizes.

In the limitations or future directions, mention that normalization could provide additional resolution, especially if serum factors alter mitochondrial density without changing per-mitochondrion function.

We agree that a change in OCR could reflect either a change in mitochondrial density or a change in the metabolic output on a per-mitochondrion basis. In our assay, we would not be able to distinguish between these two possibilities. Had we seen a difference in OCR in our study, this would have been a valuable follow-up question, but since we do not observe any differences in OCR between our groups we do not see this as an important limitation to our study.

______________________________________________________________________________________________

We thank all three reviewers for their responses and look forward to hearing their decisions or further feedback.

Audrey Ryback
